# DFT-D3 and TD-DFT Studies of the Adsorption and Sensing Behavior of Mn-Phthalocyanine toward NH_3_, PH_3_, and AsH_3_ Molecules

**DOI:** 10.3390/molecules29102168

**Published:** 2024-05-07

**Authors:** Heba Mohamed Badran, Khaled Mahmoud Eid, Hatim Omar Al-Nadary, Hussein Youssef Ammar

**Affiliations:** 1 Physics Department, College of Science & Arts, Najran University, Najran 11001, Saudi Arabia; hmbadran@nu.edu.sa; 2 Physics Department, Faculty of Education, Ain Shams University, Roxy, Cairo 11566, Egypt; khaledmahmoud@edu.asu.edu.eg; 3 STEM Pioneers Training Lab, Najran University, Najran 11001, Saudi Arabia

**Keywords:** Mn-phthalocyanine, DFT, TD-DFT, NH_3_, PH_3_, AsH_3_, adsorption, sensor

## Abstract

This study employs density functional theory (DFT) calculations at the B3LYP/6-311+g(d,p) level to investigate the interaction of XH_3_ gases (X = N, P, As) with the Mn-phthalocyanine molecule (MnPc). Grimme’s D3 dispersion correction is applied to consider long-range interactions. The adsorption behavior is explored under the influence of an external static electric field (EF) ranging from −0.514 to 0.514 V/Å. Chemical adsorption of XH_3_ molecules onto the MnPc molecule is confirmed. The adsorption results in a significant decrease in the energy gap (E_g_) of MnPc, indicating the potential alteration of its optical properties. Quantum theory of atoms in molecules (QTAIM) analysis reveals partially covalent bonds between XH_3_ and MnPc, and the charge density differenc (Δρ) calculations suggest a charge donation-back donation mechanism. The UV-vis spectrum of MnPc experiences a blue shift upon XH_3_ adsorption, highlighting MnPc’s potential as a naked-eye sensor for XH_3_ molecules. Thermodynamic calculations indicate exothermic interactions, with NH_3_/MnPc being the most stable complex. The stability of NH_3_/MnPc decreases with increasing temperature. The direction and magnitude of the applied electric field (EF) play a crucial role in determining the adsorption energy (E_ads_) for XH_3_/MnPc complexes. The E_g_ values decrease with an increasing negative EF, which suggests that the electrical conductivity (σ) and the electrical sensitivity (ΔE_g_) of the XH_3_/MnPc complexes are influenced by the magnitude and direction of the applied EF. Overall, this study provides valuable insights into the suggested promising prospects for the utilization of MnPc in sensing applications of XH_3_ gases.

## 1. Introduction

Preserving the environment stands as one of the utmost priorities for scientists today. With technological advancements permeating every facet of life, the prevalence of pollutants has escalated to a level that poses a threat to living organisms. Consequently, there arose a necessity to develop and produce nanosensors specifically designed for detecting environmentally harmful gases. Among the hydrides of the fifth group, ammonia (NH_3_), phosphine (PH_3_), and arsine (AsH_3_) merit special attention due to the hazards they pose. NH_3_ is a colorless gas with a pungent odor. NH_3_ is used in several industries, such as the production of fertilizers, pesticides, hair dyes, plastics, and the textile industry. The inhalation of ammonia can result in severe irritation to the respiratory system [1,2,3]. PH_3_ is a scentless toxic gas characterized by its spontaneous flammability in the air or when interacting with oxygen. PH_3_ has several uses; for instance, it is used to exterminate insects and rodents. It is also used to preserve agricultural crops in warehouses during storage. Its risks are due to inhalation or absorption through the skin. Inhaling PH_3_ gas causes pneumonia and respiratory poisoning and can harm the central nervous system [4,5]. AsH_3_ is a colorless gas that has a disagreeable fish-like scent. It is used in the steel industry and metal treatment. It is an extremely toxic gas. The risks caused by arsine are abdominal pain, diarrhea, dyspnea, hemolysis, and renal failure [6,7,8].

Earlier research indicates that attempts were made toward the adsorption of these hazardous gases onto the surfaces of various materials. Luo et al. [9] demonstrated that the adsorption of NH_3_, PH_3_, and AsH_3_ on graphene is weak, while the doping of graphene with La, Ce, Nd, Pm, Sm, Eu, and Gd improved the adsorption of NH_3_ and weakened the adsorption of AsH_3_. Habibi-Yangjeh et al. [4] suggested that Ta/P heptazine graphitic carbon nitride serves as a suitable sensor for PH_3_. Conversely, Ranea et al. [10] found that the V atom of V_2_O_5_ is an attractive center for NH_3_, PH_3_, and AsH_3_. Additionally, CdSe functions efficiently as a gas sensor for NH_3_, PH_3_, and AsH_3_ gases [11].

Phthalocyanines (Pcs) represent a class of aromatic macrocyclic organic compounds characterized by the molecular formula C_32_H_18_N_8_. Their distinct attributes encompass remarkable thermal and chemical stability, coupled with favorable electrical and optical properties, primarily attributed to their extensive electronic π-conjugated system [12,13].

The enumerated advantages have significantly propelled the utilization of phthalocyanines (Pcs) across diverse domains, including solar cells, semiconductors, catalysis, sensors, and tumor treatment [12,14,15,16]. Notably, research indicates that the incorporation of transition elements into phthalocyanine structures enhances their suitability for a wide range of applications. For instance, the integration of MnPc and FePc into junctions between two single-walled carbon nanotubes results in superior magnetic spin moments compared to CoPc and NiPc, thereby enhancing their performance in spintronic devices [15]. Furthermore, investigations into the magnetic properties of transition metal phthalocyanine sheets (TM = Cr-Zn) reveal that only MnPc exhibits ferromagnetic behavior [17]. Doping Pcs with Co and Mn has been shown to heighten their redox activity, a crucial aspect for electrochemical applications [16]. Given these findings, it can be reasonably anticipated that grafting phthalocyanine with a transition element, particularly Mn, will induce notable changes in its properties as a gas sensor.

To the best of our knowledge, the application of MnPc as an adsorbent or sensor for NH3, PH3, and AsH3 gases has not been explored previously. This study is designed to investigate the adsorption properties of NH_3_, PH_3_, and AsH_3_ gases on the MnPc molecule. Additionally, we will explore the impact of an external static electric field on these adsorption characteristics.

## 2. Results and Discussions

In this study, we examined the adsorption of XH_3_ gases (where X = N, P, As) on a MnPc molecule. Initially, we focused on determining the most energetically stable structures of both the individual XH_3_ and MnPc molecules. This investigation aimed to understand the influence of the Mn atom on the properties of Pc. Subsequently, we investigated the most energetically stable structures of the XH_3_/Pc complexes.

### 2.1. Properties of XH_3_ Adsorbates and the MnPc Adsorbent

**Figure 1:**
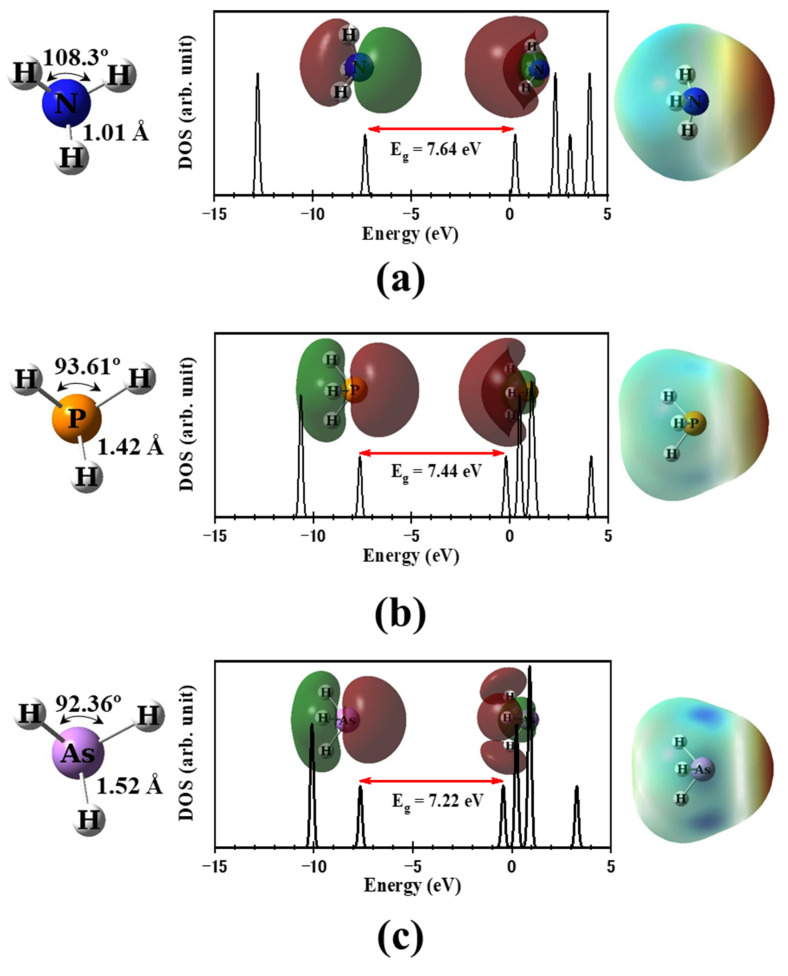
Geometrical structure, DOS (HOMO and LUMO are inserted), and MESP for (**a**) NH_3_, (**b**) PH_3_, and (**c**) AsH_3_. Red and blue colors represent negative and positive values of MESP, respectively.

Figure 1 displays the geometrical structure, density of states (DOS), highest occupied molecular orbital (HOMO), lowest unoccupied molecular orbital (LUMO), and the molecular electrostatic potential (MESP) for XH_3_ molecules. The X–H bond lengths and H–X–H angles were determined as follows: NH_3_ (1.01 Å, 108.3°), PH_3_ (1.42 Å, 93.61°), and AsH_3_ (1.52 Å, 92.36°), which are consistent with previous findings [2,3,18,19]. The MESP analysis reveals a negative electrostatic potential around the X atom, indicating the presence of a 2p electron lone pair. This suggests that the X atom functions as a nucleophilic center. Moreover, the calculated electrical dipole moments (D) were found to be 1.673, 0.823, and 0.345 Debye for NH_3_, PH_3_, and AsH_3_, respectively, which aligns with the findings of Zhang et al. [18]. Consequently, the chemical reactivity of a molecule increases with its dipole moment [14]. Therefore, one can anticipate the following trend in chemical reactivity: NH_3_ > PH_3_ > AsH_3_.

**Figure 2:**
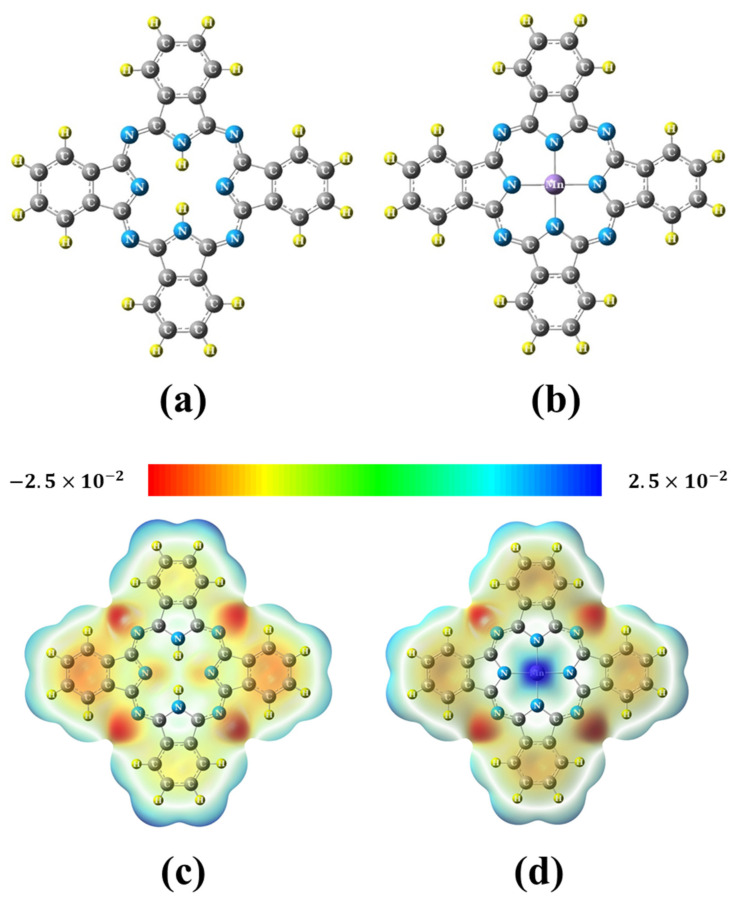
(**a**,**b**) The optimized structures and (**c**,**d**) molecular electrostatic potential (MESP) in atomic units for Pc and MnPc, respectively.

**Figure 3:**
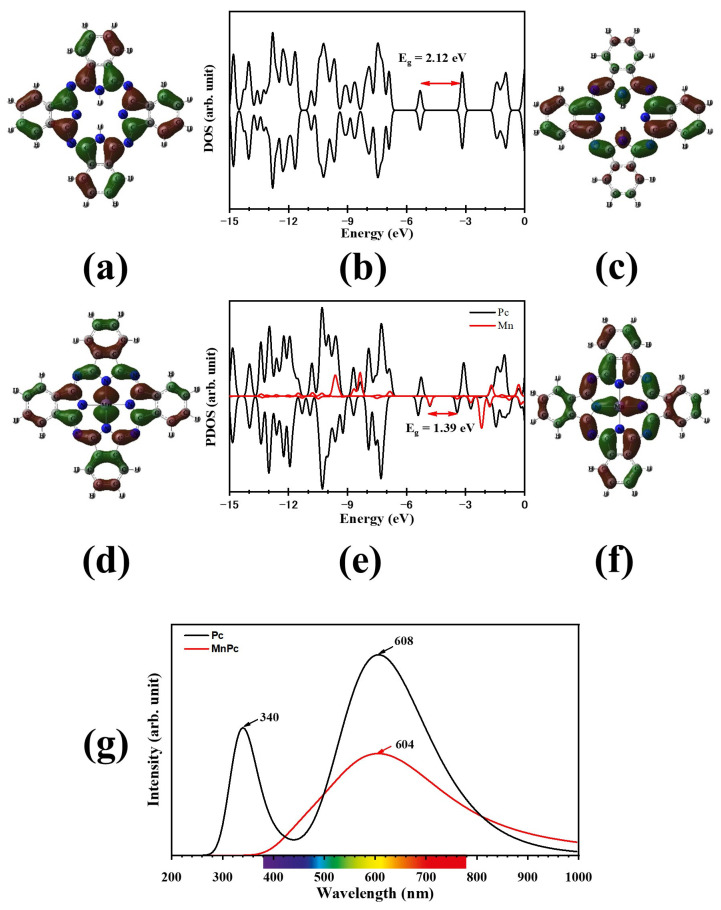
(**a**–**c**) HOMO, DOS, and LUMO for Pc, (**d**–**f**) HOMO, PDOS, and LUMO for MnPc, and (**g**) UV-vis spectra for Pc and MnPc, respectively.

Figure 2 illustrates the optimized structures and MESP for Pc and MnPc, while their electronic properties are summarized in Table 1. It was observed that in MnPc, the Mn–N bond length was determined to be 1.958 Å and the N–Mn–N angle was found to be 90°, which is consistent with previous theoretical and experimental studies [15,20,21]. The calculated band gap (E_g_) values indicate that both Pc and MnPc are semiconductors, with the presence of the Mn atom leading to a 34.5% reduction in the E_g_ value of the Pc molecule. Furthermore, the binding energy (E_b_) for MnPc was more negative compared to that of Pc, implying that the Mn atom enhances the stability of the MnPc molecule. This can be attributed to intramolecular partial charge transfer (PCT) occurring from the Mn atom to the rest of the molecule, resulting in a positive charge of 0.954 e accumulating on the Mn atom. Moreover, the MnPc molecule exhibits lower values of E_g_, IP, and η, while displaying higher values of E_f_ and ω compared to the Pc molecule. These characteristics indicate that the MnPc molecule is more reactive than the Pc molecule. Additionally, the positive charge on the Mn atom results in a positive electrostatic potential surrounding it, as depicted in Figure 2d. Consequently, the Mn atom can be considered an electrophilic site. It is worth noting that the Pc and MnPc molecules do not exhibit noticeable values of the electrical dipole moment (D) due to the symmetrical distribution of electrical charges on the atoms within these molecules. Figure 3b,e depict the DOS and PDOS for Pc and MnPc molecules, respectively. It is evident that the presence of the Mn atom causes the HOMO to shift upwards by 0.507 eV, while the LUMO is shifted downwards by 0.225 eV. Consequently, the E_g_ value of the Pc molecule decreases from 2.120 eV to 1.388 eV in the MnPc molecule, which is consistent with previous research [21,22].

Furthermore, the UV-vis spectra of Pc and MnPc molecules are displayed in Figure 3g. The Pc molecule exhibits two absorption peaks, known as the Soret band and Q band, at wavelengths of 340 nm and 608 nm, respectively. These findings align with previous studies [22,23,24]. On the other hand, the MnPc molecule demonstrates a broad absorption peak at 604 nm.

### 2.2. Adsorption of XH_3_ on MnPc

**Figure 4:**
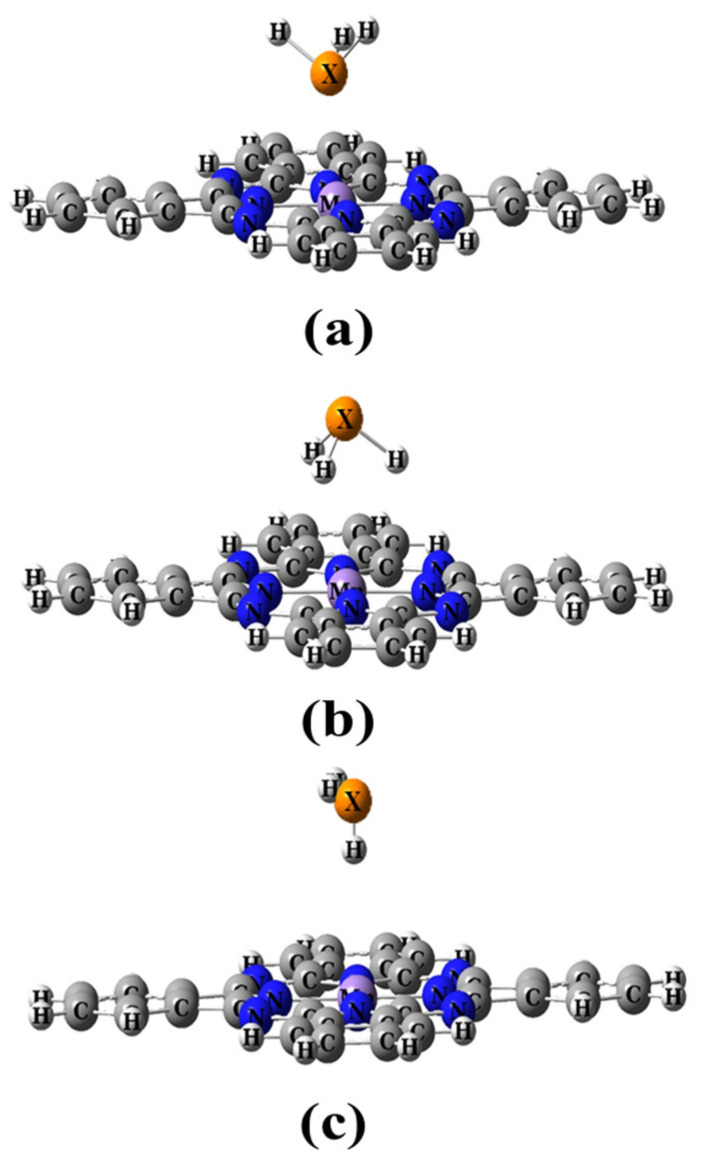
The proposed adsorption modes for the XH_3_ molecule on the MnPc molecule (**a**) mode 1, (**b**) mode 2, and (**c**) mode 3.

**Figure 5:**
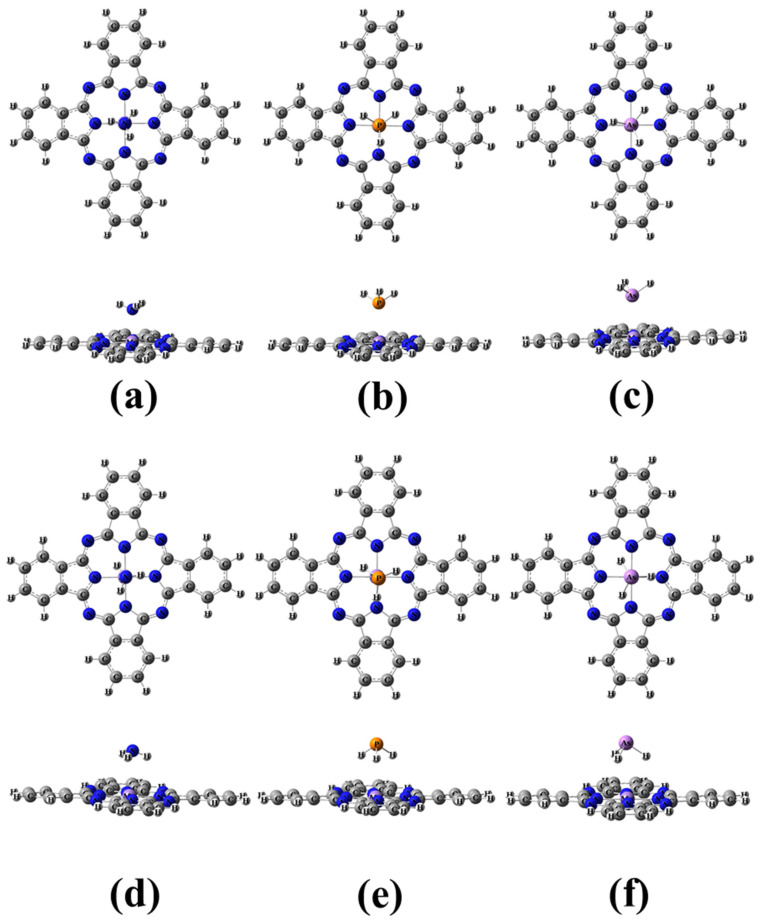
Top and side views of the optimized structures (**a**–**c**) for adsorption mode 1 and (**d**–**f**) for adsorption mode 2 for NH_3_/MnPc, PH_3_/MnPc, and AsH_3_/MnPc, respectively.

The adsorption of XH_3_ molecules on the Mn site of the MnPc molecule was investigated in three different adsorption modes, as shown in Figure 4. All modes undergo full optimization, and it was found that modes 1 and 2 resulted in energetically stable complexes. Interestingly, mode 3 reoriented to yield the same complexes as mode 2. Top and side views of the optimized complexes for adsorption modes 1 and 2 are presented in Figure 5. The adsorption properties for modes 1 and 2 are summarized in Table 2.

In accordance with previous studies [25,26,27], it is well-established that chemisorption is characterized by high adsorption energy ($\left|E_{ads}\right|$ ≥ 0.2 eV). Therefore, based on the obtained XH_3_/MnPc complexes in adsorption modes 1 and 2, it can be concluded that the XH_3_ molecule undergoes chemical adsorption on the MnPc molecule. It is important to note that a more negative E_ads_ value indicates a higher degree of adsorption. For adsorption mode 1, the trend of adsorption strength was observed as NH_3_/MnPc > PH_3_/MnPc > AsH_3_/MnPc complexes, indicating that NH_3_ exhibited the highest adsorption strength, followed by PH_3_ and AsH_3_. On the other hand, for adsorption mode 2, the trend was NH_3_/MnPc < PH_3_/MnPc < AsH_3_/MnPc, suggesting that AsH_3_ exhibited the highest adsorption strength, followed by PH_3_ and NH_3_. Furthermore, it can be observed that the adsorption strength in mode 1 was higher compared to mode 2. The distances between the X atom and the Mn adsorbing site (d_X-Mn_) followed the trend of NH_3_/MnPc < PH_3_/MnPc < AsH_3_/MnPc for both adsorption modes. This indicates that NH_3_ exhibited the closest proximity to the Mn adsorbing site, followed by PH_3_ and AsH_3_, in both modes.

Furthermore, the adsorption of XH_3_ molecules resulted in a decrease in the E_g_ value of the MnPc molecule. Specifically, for adsorption mode 1, the E_g_ value decreased to 86.2%, 91.6%, and 92.8% for NH_3_/MnPc, PH_3_/MnPc, and AsH_3_/MnPc, respectively. For adsorption mode 2, the E_g_ value decreased to 96.2%, 98.2%, and 97.9% for NH_3_/MnPc, PH_3_/MnPc, and AsH_3_/MnPc, respectively. In other words, adsorption mode 1 resulted in a greater reduction in the E_g_ value compared to adsorption mode 2. Notably, the largest decrease in the E_g_ value was observed for the NH_3_/MnPc complex in adsorption mode 1. Additionally, the adsorption of XH_3_ molecules led to an increase in the dipole moment. Specifically, for adsorption mode 1, the dipole moment increased to 3.130, 2.083, and 1.576 Debye for NH_3_/MnPc, PH_3_/MnPc, and AsH_3_/MnPc, respectively. For adsorption mode 2, the dipole moment increased to 0.949, 0.228, and 0.213 Debye for NH_3_/MnPc, PH_3_/MnPc, and AsH_3_/MnPc, respectively.

**Figure 6:**
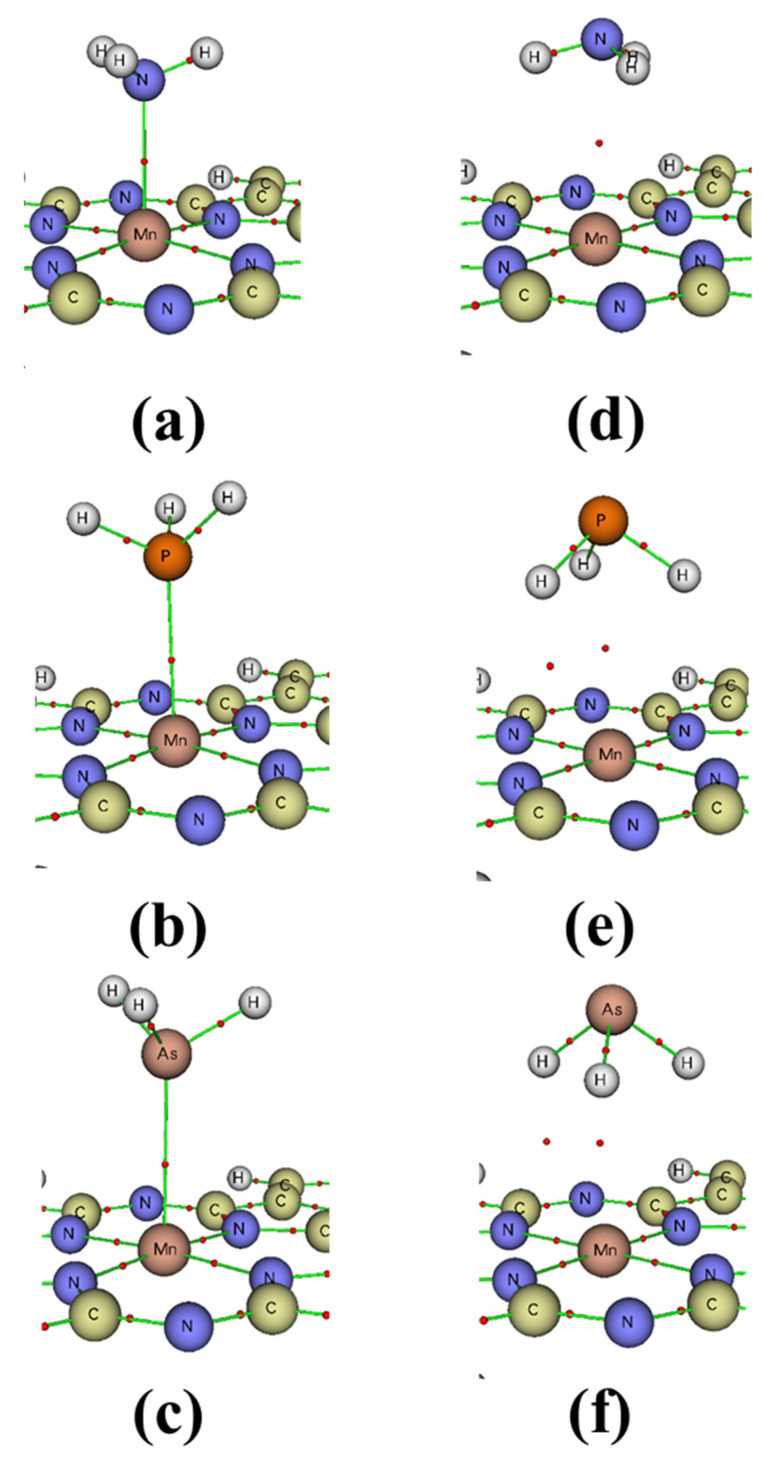
Bond critical points of type (3, −1): (**a**–**c**) for adsorption mode 1 and (**d**–**f**) for adsorption mode 2 for NH_3_/MnPc, PH_3_/MnPc, and AsH_3_/MnPc, respectively.

The results obtained can be further analyzed using several methods, including QTAIM, electrostatic potential (ESP), NBO atomic charge, charge density difference (∆ρ), and PDOS. The QTAIM analysis is particularly useful for understanding the nature of interactions [28,29,30]. Previous studies have established certain characteristics for different types of interactions, such as van der Waals interactions, weak hydrogen bonds, and ionic bonds. These characteristics include −G(r)/V(r) > 1, H(r) > 0, and ∇^2^ρ > 0. Strong interactions are classified by ∇^2^ρ > 10^–1^ au, while weak interactions have ∇^2^ρ < 10^–1^ au. Partially covalent bonds are characterized by ∇^2^ρ > 0 and 0.5 < –G(r)/V(r) < 1. Therefore, the QTAIM theory can be employed to analyze the topological parameters of the bond critical points (BCP) of type (3, –1) formed between the XH_3_ and MnPc molecules. The BCPs, as depicted in Figure 6, and their corresponding parameters are summarized in Table 3. The analysis reveals that in adsorption mode 1, only one BCP exists between the X atom of the XH_3_ molecule and the Mn atom of the MnPc molecule. This BCP exhibits a ∇^2^ρ value greater than zero, specifically 0.171, 0.064, and 0.046 au for NH_3_/MnPc, PH_3_/MnPc, and AsH_3_/MnPc complexes, respectively. Furthermore, the −G(r)/V(r) ratio for these BCPs is greater than 0.5 but less than 1, specifically 0.952, 0.813, and 0.833 for NH_3_/MnPc, PH_3_/MnPc, and AsH_3_/MnPc complexes, respectively. As a result, these BCPs are classified as partially covalent bonds. Moreover, the ∇^2^ρ values indicate that the interaction in the NH_3_/MnPc complex is characterized as strong, while the interactions in the PH_3_/MnPc and AsH_3_/MnPc complexes are classified as weak.

In adsorption mode 2, the NH_3_/MnPc complex exhibits one BCP between the N atom of the NH_3_ molecule and the Mn atom of the MnPc molecule, with a ∇^2^ρ value of 0.030 au and a −G(r)/V(r) ratio of 0.897. This BCP is classified as a weak, partially covalent bond. On the other hand, for the PH_3_/MnPc and AsH_3_/MnPc complexes, two BCPs are observed between the XH_3_ molecule and the MnPc molecule. The first BCP (X–Mn) is formed between the X atom of the XH_3_ molecule and the Mn atom of the MnPc molecule, while the second BCP (H–N) is formed between an H atom of the XH_3_ molecule and an N atom of the MnPc molecule. These BCPs have positive ∇^2^ρ values of less than 10^–1^ and −G(r)/V(r) ratios greater than 1. Consequently, the first BCPs (X–Mn) are classified as van der Waals interactions, while the second BCPs (H–N) are classified as weak hydrogen bonds.

**Figure 7:**
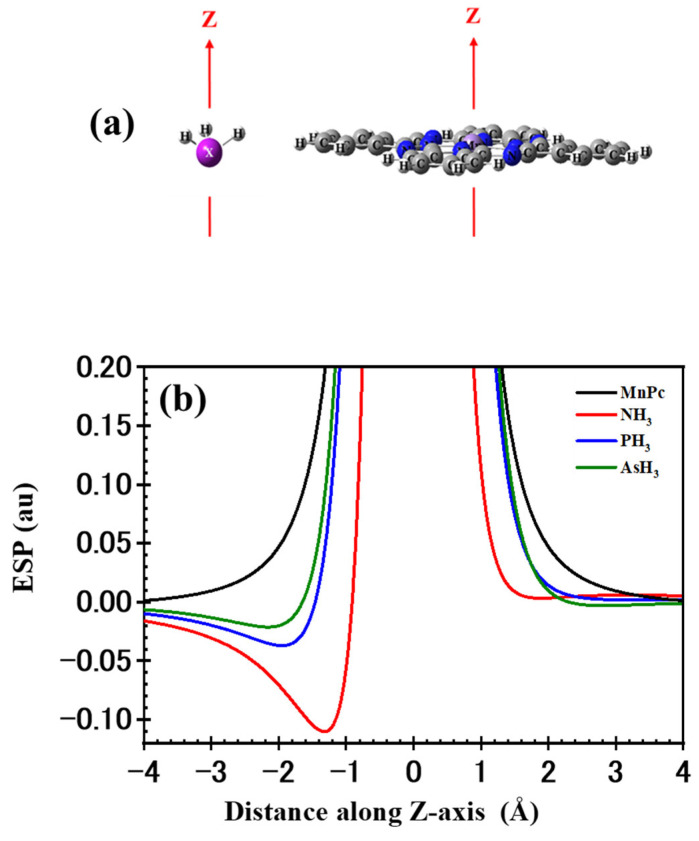
(**a**) Orientation and (**b**) electrostatic potential (ESP) for XH_3_ and MnPc molecules.

Figure 7 illustrates the ESP for the MnPc and XH_3_ molecules. The ESP was calculated along the *Z*-axis, with the X and Mn atoms positioned at the origin point. The ESP for the MnPc molecule appears positive and symmetric around the Mn atom along the *Z*-axis. In contrast, the ESP for the XH_3_ molecule exhibits an asymmetric pattern along the *Z*-axis.

In the −Z direction, the ESP curves for NH_3_, PH_3_, and AsH_3_ display minimum negative values of −0.110, −0.037, and −0.021 au, respectively, occurring at distances of –1.303, −1.949, and −2.152 Å from the origin. On the other hand, in the +Z direction, the ESP curves have minimum values of 0.003, 0.002, and −0.003 au at distances of 1.887 Å, 3.373 Å, and 2.828 Å, respectively. Notably, the minima of the curves in the –Z direction are lower than those in the +Z direction. This discrepancy arises from the presence of the X atom’s electron lone pair in the –Z direction. These observations may help explain the stronger interaction observed for adsorption in mode 1 compared to mode 2, as well as the trend observed in the adsorption behavior. To analyze partial charge transfer (PCT), NBO atomic charges were computed. In all the examined adsorption structures, the XH_3_ molecule undergoes a positive charge, suggesting a transfer of charge from the XH_3_ molecule to the MnPc molecule. This PCT is more significant in adsorption mode 1 than in adsorption mode 2. Consequently, it is reasonable to anticipate that the PCT contributes to the reinforcement of adsorption mode 1. In addition, for adsorption mode 1, the XH_3_ molecule experiences a loss of charges ($Q_{XH_{3}}$) of 0.177, 0.324, and 0.226 e, while the Mn adsorbing site gains charges of 0.127, 0.125, and 0.187 e for NH_3_/MnPc, PH_3_/MnPc, and AsH_3_/MnPc, respectively. This implies that the XH_3_ molecule not only transfers charge to the adsorbing site but also to the other atoms of the MnPc molecule. Specifically, the XH_3_ molecule loses more charge than what is gained by the Mn adsorbing site.

**Figure 8:**
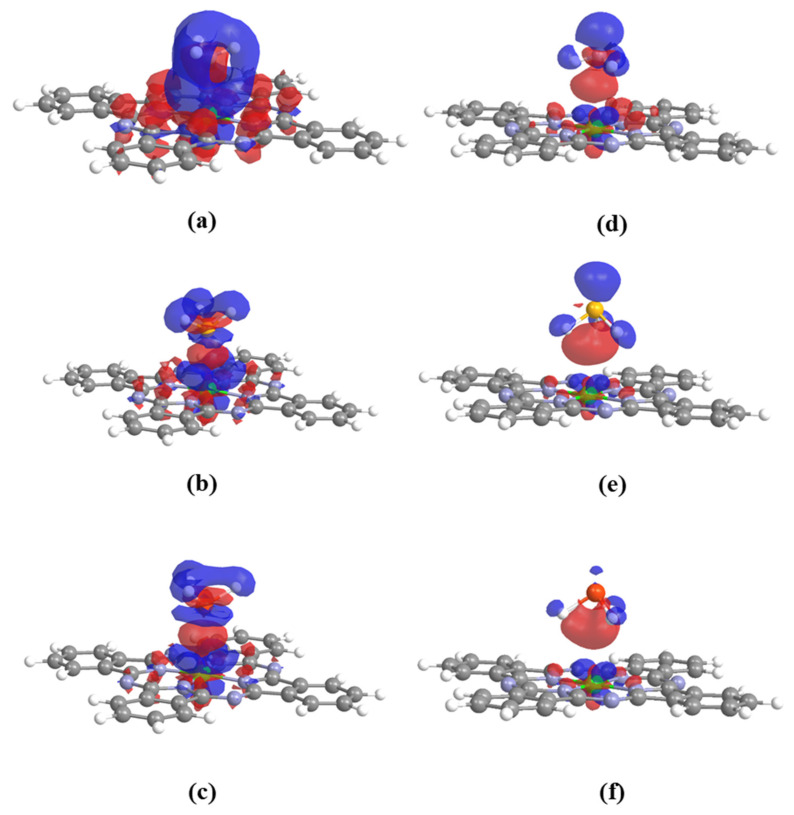
Charge density difference (Δρ) at 0.001 au isovalue (**a**–**c**) for adsorption mode 1 and (**d**–**f**) for adsorption mode 2 for NH_3_/MnPc, PH_3_/MnPc, and AsH_3_/MnPc, respectively. Red and blue colors refer to negative and positive Δρ values.

It is important to note that the trend of $Q_{XH_{3}}$ does not necessarily align with the trend of E_ads_, indicating the presence of another mechanism influencing the adsorption process. To further elucidate this, the charge density differences (Δρ) for the XH_3_/MnPc complexes were analyzed and depicted in Figure 8. The Δρ values exhibit both positive (blue) and negative (red) regions for both the XH_3_ adsorbate and MnPc substrate. This suggests that charge transfer occurs in two directions, from the XH_3_ molecule to the MnPc molecule and vice versa. Consequently, a charge donation-back donation mechanism is proposed for the adsorption process. Furthermore, Figure 8 illustrates that the Δρ values for adsorption mode 1 are higher than those for adsorption mode 2, which corresponds well with the adsorption energies described in Table 2. Lastly, as a result of the adsorption, a redistribution of charges between the XH_3_ and MnPc molecules occurs, leading to an increase in the electric dipole moment values of the XH_3_/MnPc complexes.

**Figure 9:**
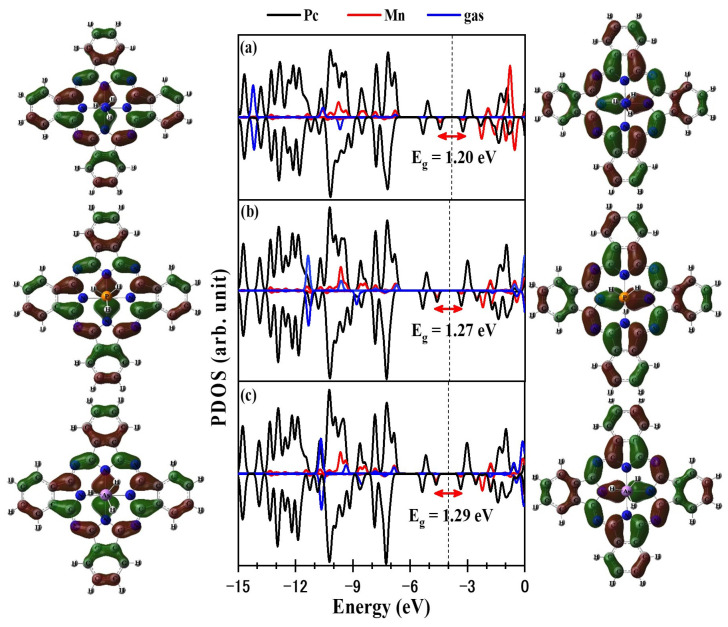
HOMO, PDOS, and LUMO for adsorption mode 1 (**a**) NH_3_/MnPc, (**b**) PH_3_/MnPc, and (**c**) AsH_3_/MnPc. The dashed line refers to the Fermi level.

**Figure 10:**
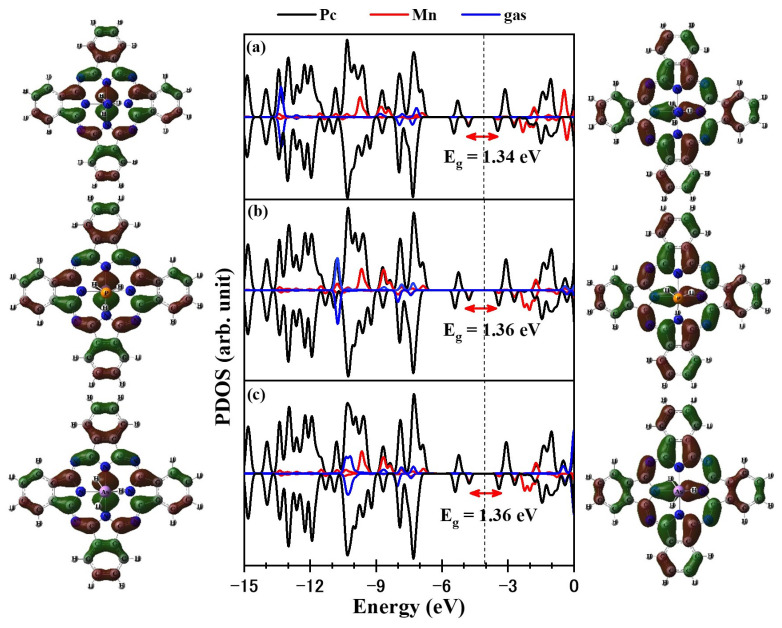
HOMO, PDOS, and LUMO for adsorption mode 2 (**a**) NH_3_/MnPc, (**b**) PH_3_/MnPc, and (**c**) AsH_3_/MnPc. The dashed line refers to the Fermi level.

The partial densities of states (PDOS) for the XH_3_/MnPc complexes are presented in Figure 9 for adsorption mode 1 and Figure 10 for adsorption mode 2. By comparing the DOS of the XH_3_ molecule prior to adsorption (Figure 1) with those after adsorption (Figure 9 and Figure 10), significant changes are evident, indicating the interaction between the XH_3_ molecule and the MnPc molecule. As a result of this interaction, the HOMO of the MnPc molecule undergoes a shift in energy. Specifically, the HOMO is shifted from −4.798 eV to −5.079, −5.136, and −5.163 eV for the NH_3_/MnPc, PH_3_/MnPc, and AsH_3_/MnPc complexes, respectively. This shift in energy reflects the influence of the XH_3_ molecule on the electron density distribution and electronic structure of the MnPc molecule. Similarly, the LUMO of the MnPc molecule experiences an energy shift following the interaction with the XH_3_ molecule. The LUMO is shifted from −3.410 eV to −3.230, −3.312, and −3.340 eV for the NH_3_/MnPc, PH_3_/MnPc, and AsH_3_/MnPc complexes, respectively. This shift in energy suggests changes in the accessibility of the LUMO for electron transfer or participation in chemical reactions.

Overall, the observed shifts in the HOMO and LUMO energies of the MnPc molecule indicate the modification of its electronic structure due to the interaction with the XH_3_ molecule in the XH_3_/MnPc complexes. These changes in the DOS further highlight the influence of the XH_3_ adsorbate on the electronic properties of the MnPc substrate.

Consequently, for adsorption mode 1, the shifts in the HOMO and LUMO energies lead to a narrowing of the bandgap (E_g_) for the NH_3_/MnPc, PH_3_/MnPc, and AsH_3_/MnPc complexes by 13.80%, 8.43%, and 7.21%, respectively. In contrast, for adsorption mode 2, the shifts in the HOMO and LUMO energies are not as significant, resulting in a smaller reduction in the E_g_ values. Specifically, the E_g_ values for the NH_3_/MnPc, PH_3_/MnPc, and AsH_3_/MnPc complexes are slightly narrowed by 3.78%, 1.82%, and 2.08%, respectively.

Furthermore, Equation (1) represents the relationship between the electrical conductivity (σ) and the HOMO-LUMO gap (E_g_). This equation suggests that a smaller E_g_ corresponds to a higher electrical conductivity [31,32,33,34,35,36].

(1) $$\sigma =AT^{3/2}e^{-E_{g}/2KT}$$

where A is a constant, K is Boltzmann’s constant, and T is the temperature. As a result, it is reasonable to assume that the XH_3_ molecule’s adsorption will raise the MnPc molecule’s electrical conductivity. Furthermore, the increase in σ in the case of the adsorption mode 1 is greater than in the case of the adsorption mode 2, and in the case of NH_3_, it is greater than in the case of PH_3_ and AsH_3_. Consequently, the MnPc molecule exhibits a higher sensitivity for NH_3_ molecules than PH_3_ and AsH_3_ molecules. Our results for mode 1 clarify that MnPc may be useful for XH_3_ detection, especially NH_3_ detection.

### 2.3. UV-Vis Spectra Analysis

**Figure 11:**
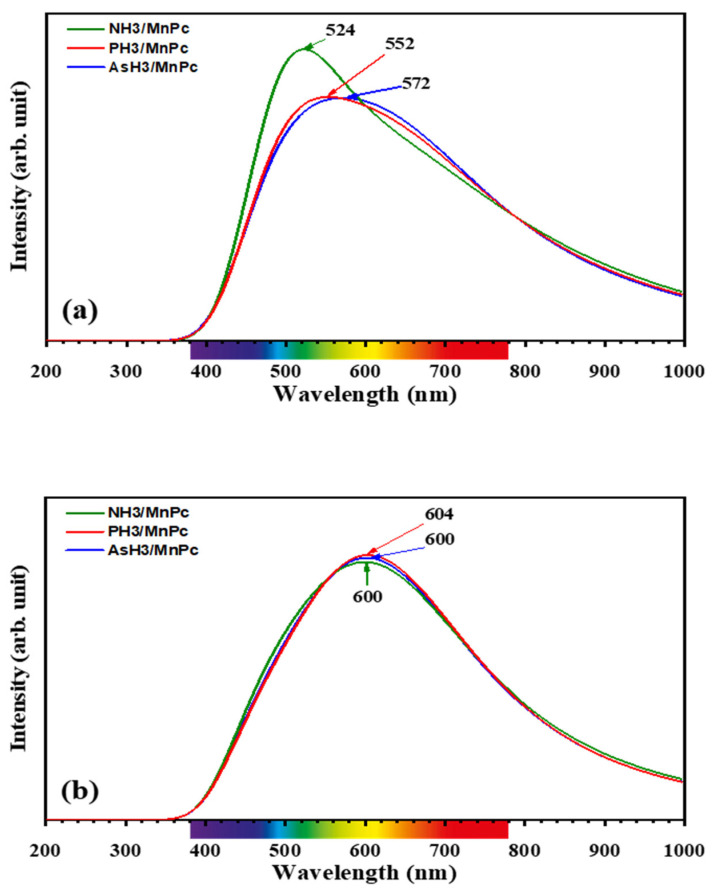
UV-vis spectra for XH_3_/Pc complexes for (**a**) adsorption mode 1 and (**b**) adsorption mode 2.

The influence of XH_3_ adsorption on the UV-vis spectrum of the MnPc molecule was investigated for adsorption modes 1 and 2. TD-DFT calculations were performed on the optimized structures of XH_3_/MnPc complexes to predict the UV-vis absorption spectra. In Figure 11a,b, the UV-vis spectra for XH_3_/MnPc complexes in adsorption modes 1 and 2 are presented, respectively.

For adsorption mode 1, the NH3/MnPc, PH3/MnPc, and AsH3/MnPc complexes exhibit maximum absorption wavelength peaks (λ_max_) in the visible region at 524, 552, and 572 nm, respectively. In contrast, Figure 3g illustrates that the λ_max_ of the MnPc molecule is situated at 604 nm. This indicates that the adsorption of XH_3_ molecules induces a blue shift in the UV-vis spectrum of MnPc. Consequently, the color of the MnPc molecule may be altered by the adsorption of XH_3_ molecules, suggesting the potential utility of MnPc as a naked-eye sensor for the investigated XH_3_ molecules.

Conversely, for adsorption mode 2, the NH_3_/MnPc, PH_3_/MnPc, and AsH_3_/MnPc complexes display λ_max_ in the visible region at 604, 600, and 600 nm, respectively. In this case, XH_3_ molecules exhibit no significant impact on the UV-vis spectrum of MnPc.

### 2.4. Thermodynamic Analysis

Given that the majority of gas sensors operate at temperatures below 800 K [37,38], thermodynamic calculations were conducted for the XH_3_ molecules, MnPc substrate, and XH_3_/MnPc complexes within the temperature range of 300 to 800 K. As adsorption mode 1 exhibited the most pronounced impact on the electrical and optical properties of the MnPc molecule, subsequent discussions will focus on this particular adsorption mode.

**Figure 12:**
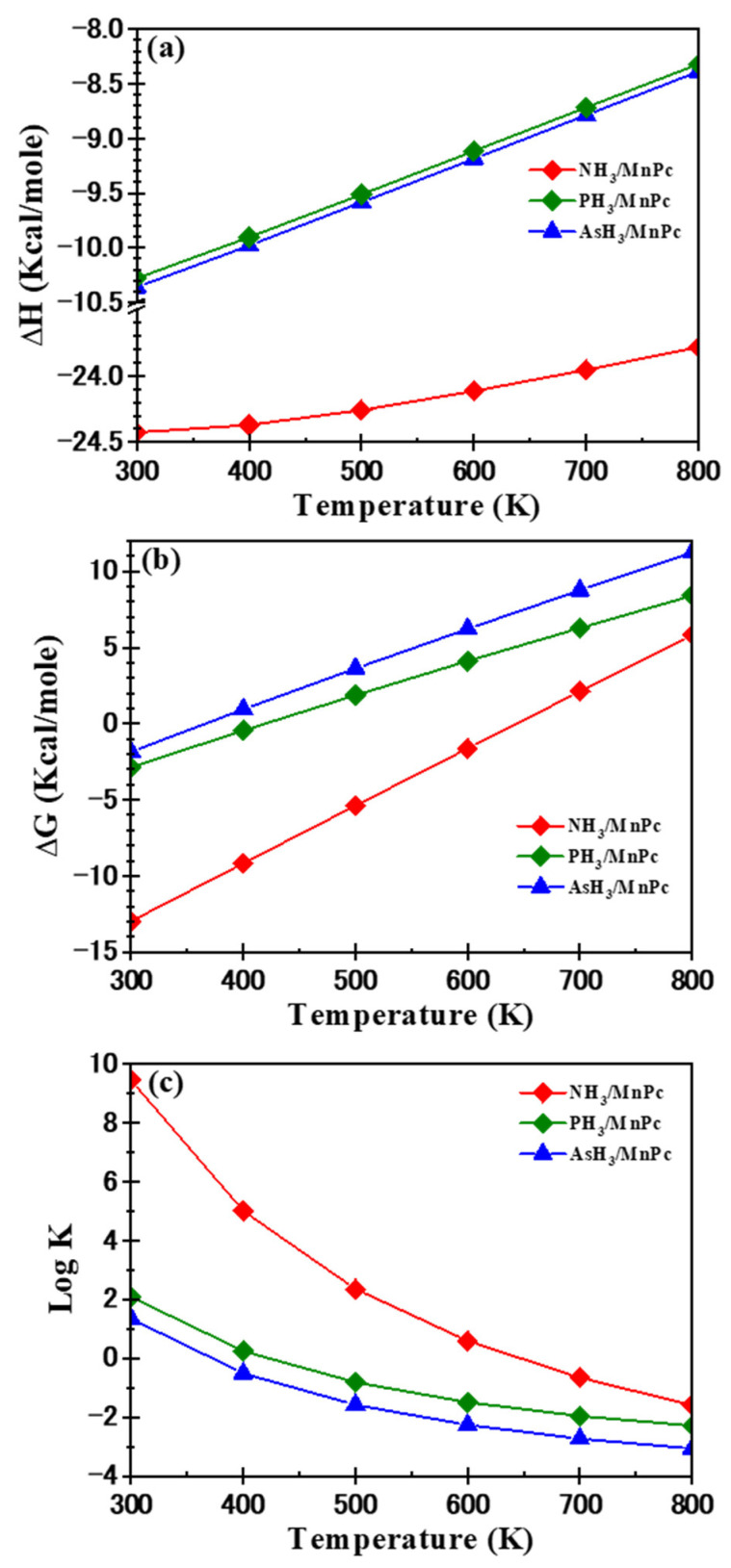
Enthalpy difference (ΔH), Gibbs free energy difference (ΔG), and the logarithm of the thermodynamic adsorption equilibrium constant (log K) for adsorption mode 1 (**a**) NH_3_/Pc, (**b**) PH_3_/Pc, and (**c**) AsH_3_/Pc.

The thermodynamic parameters, including enthalpy difference (ΔH) and free energy difference (ΔG), play a crucial role in characterizing the strength and spontaneity of gas adsorption. In the case of XH_3_/MnPc complexes, ΔH was computed using Equation (10) and is illustrated as a function of temperature (T) in Figure 12a. Negative ΔH values are indicative of an exothermic reaction, and a more negative ΔH signifies greater stability of the products. Figure 12a demonstrates that for XH_3_/MnPc complexes, the ΔH values exhibit negativity across the entire temperature range under consideration. Furthermore, as temperature increases, the ΔH values become less negative. Notably, at a specific temperature, the ΔH value for the NH_3_/MnPc complex is more negative compared to the PH_3_/MnPc and AsH_3_/MnPc complexes. This observation suggests that the interaction between the XH_3_ molecule and the MnPc molecule is exothermic. Specifically, the NH_3_/MnPc complex is more stable than the PH_3_/MnPc and AsH_3_/MnPc complexes. Additionally, the stability of NH_3_/MnPc complexes decreases with an increase in temperature.

The ΔG values for the XH_3_/MnPc complexes were determined using Equation (11) and are depicted as a function of temperature in Figure 12b. Negative and positive ΔG values signify spontaneous and non-spontaneous reactions, respectively, with low negative ΔG values suggesting a potential for reversing the reaction [39,40,41]. Figure 12b shows a linear increase in ΔG values with temperature. At room temperature (300 K), all investigated complexes display negative ΔG values. However, beyond 300, 400, and 600 K for AsH_3_/MnPc, PH_3_/MnPc, and NH_3_/MnPc complexes, respectively, ΔG values turn positive, indicating a non-spontaneous adsorption process. Additionally, at T = 300 K, the ΔG value for the NH_3_/MnPc complex is more negative than that for the PH_3_/MnPc and AsH_3_/MnPc complexes. Consequently, the adsorption process can be more easily reversed for the PH_3_/MnPc and AsH_3_/MnPc complexes than for the NH_3_/MnPc complex.

The thermodynamic adsorption equilibrium constant (K) for the adsorption process was calculated using Equation (12), and log K was plotted against temperature, as shown in Figure 12c. K serves as a crucial parameter in assessing the strength and spontaneity of the adsorption process, with higher K values indicating stronger adsorption [42]. Furthermore, K values greater than 1 suggest spontaneous adsorption, while values less than 1 indicate non-spontaneous adsorption. Observing Figure 12c, it is evident that log K for the NH_3_/MnPc complex surpasses that of the PH_3_/MnPc and AsH_3_/MnPc complexes. Consequently, the adsorption of the NH_3_ molecule is stronger compared to the adsorption of PH_3_ and AsH_3_ molecules. Additionally, for the examined XH_3_/MnPc complexes, log K decreases as temperature increases, signifying that elevated temperatures diminish the ability of the MnPc molecule to adsorb XH_3_ molecules. Moreover, beyond 300, 400, and 600 K for AsH_3_/MnPc, PH_3_/MnPc, and NH_3_/MnPc complexes, respectively, the K value falls below 1 (log K < 0), highlighting non-spontaneous adsorption.

### 2.5. Effect of the EF on the Adsorption of XH_3_ on MnPc

**Figure 13:**
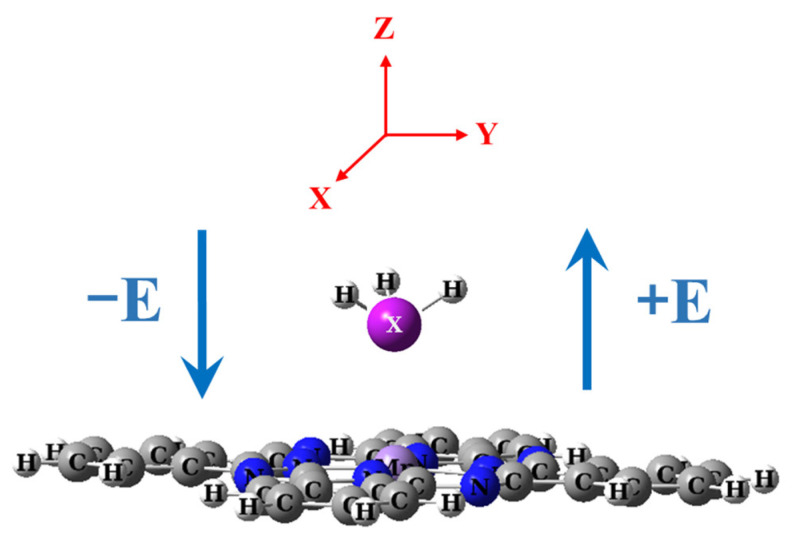
Electric field direction relative to the XH_3_/MnPc complex (X = N, P, and As).

**Figure 14:**
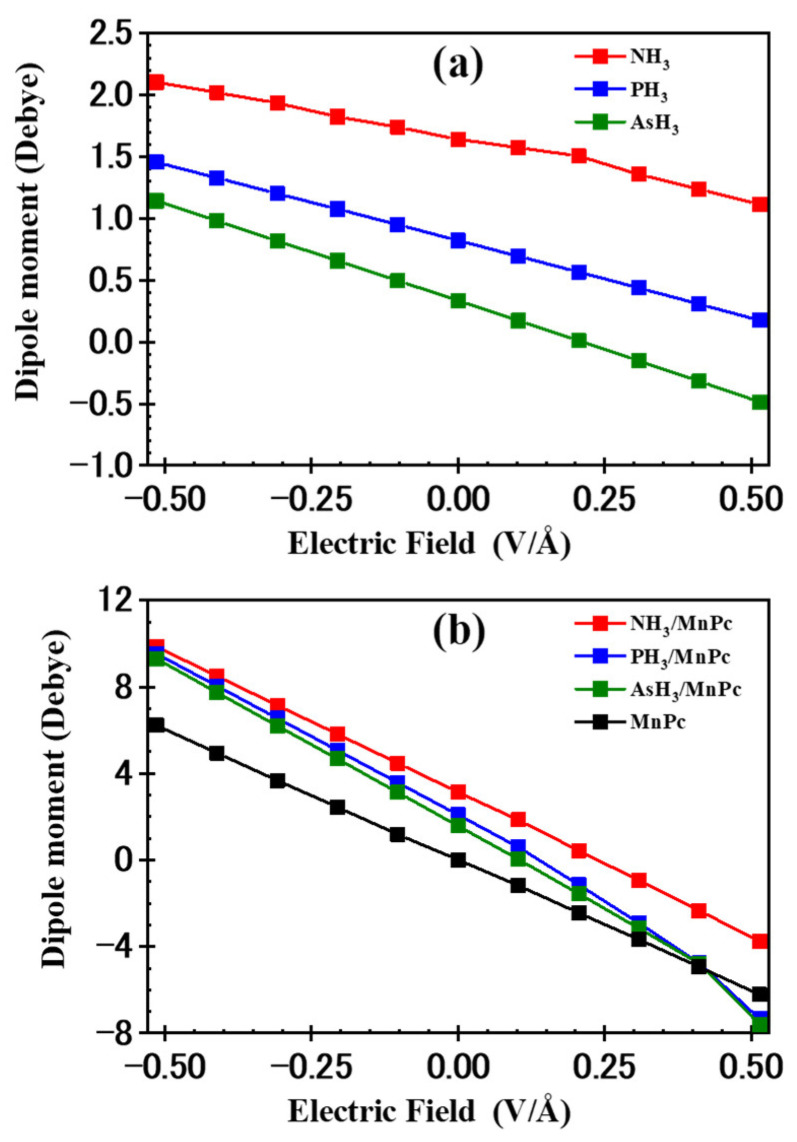
The dipole moment vs. the electric field for (**a**) free XH_3_ gases and (**b**) MnPc and XH_3_/MnPc complexes.

This study explored the adsorption properties under the influence of an external static electric field (EF), applied within the range of −0.514 to 0.514 V/Å with increments of 0.125 V/Å along the axis perpendicular to the MnPc molecule’s plane, as depicted in Figure 13. At each EF step, both XH_3_ and MnPc molecules, as well as XH_3_/MnPc complexes, undergo full optimization. The dipole moment plotted against the electric field is illustrated in Figure 14a for free XH_3_ gases and Figure 14b for MnPc and XH_3_/MnPc complexes.

**Figure 15:**
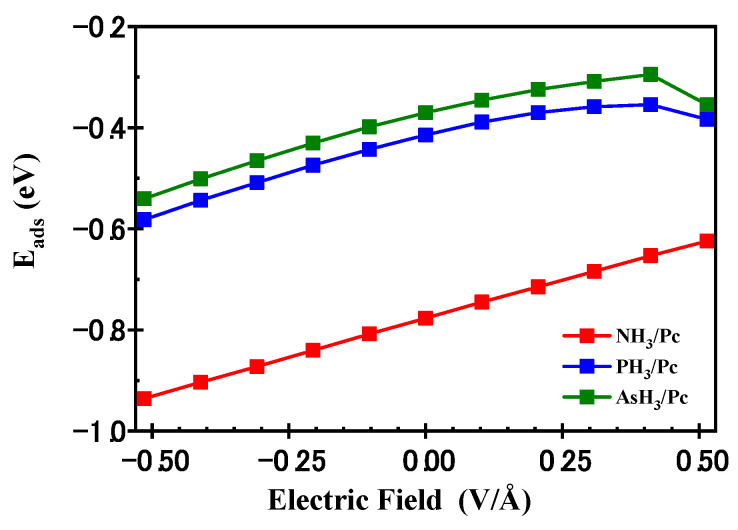
The adsorption energies (E_ads_) vs. the electric field for XH_3_/MnPc complexes.

Figure 14 presents the z-component of the dipole moment for free XH_3_ molecules, the MnPc molecule, and XH_3_/MnPc complexes in response to the applied electric field (EF) along the *Z*-axis. Notably, the z-component of the dipole moment is considered for this analysis. In Figure 14a, it is evident that the dipole moment of XH_3_ molecules increases with the magnitude of the EF in the negative direction and decreases with increasing the EF in the positive direction. Furthermore, the dipole moment follows the trend NH_3_ > PH_3_ > AsH_3_. Moving to Figure 14b, the dipole moment of the MnPc molecule increases with the magnitude of the EF in the negative direction, while it decreases with increasing the EF in the positive direction. The chemical reactivity of a material with its surrounding environment tends to increase with a higher dipole moment [14]. The dipole moments of both XH_3_ and MnPc molecules are enhanced by negative EF values, leading to an enhancement of the adsorption energy (refer to Figure 15). Conversely, positive EF values result in a decrease in the dipole moment for both the MnPc and XH_3_ molecules, leading to inhibition of the adsorption energy (refer to Figure 15). Therefore, the direction and magnitude of the applied EF play a crucial role in determining the adsorption energy (E_ads_) for XH_3_/MnPc complexes.

**Figure 16:**
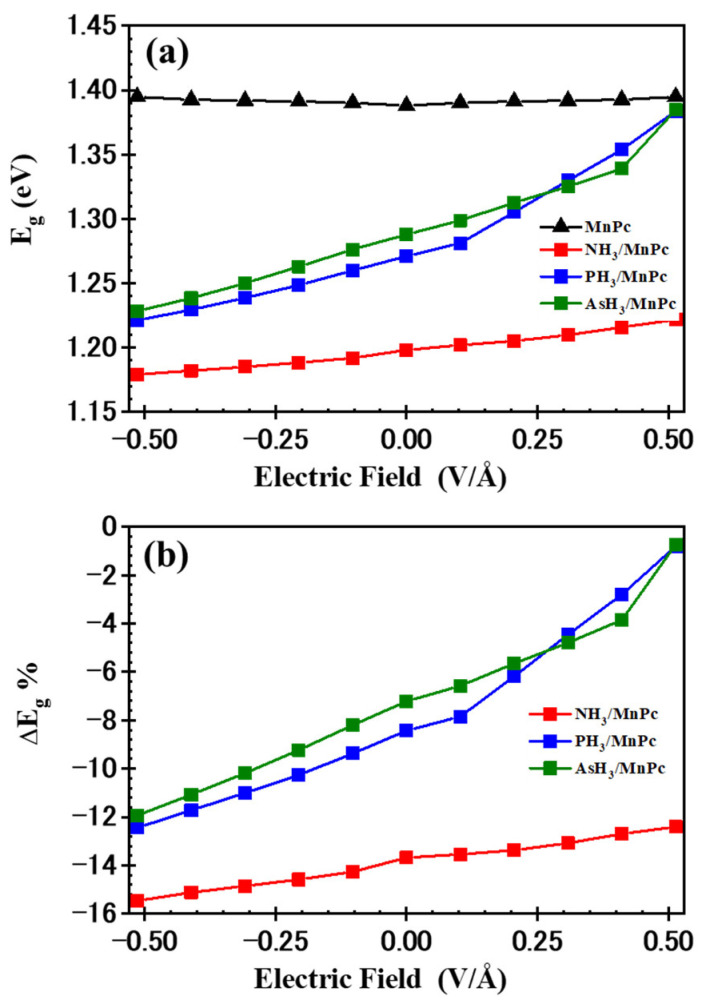
(**a**) E_g_ and (**b**) ΔE_g_% versus the electric field for MnPc and XH_3_/MnPc complexes.

The impact of the EF on the E_g_ values for the MnPc molecule and XH_3_/MnPc complexes is illustrated in Figure 16a. It is observed that the E_g_ value for the MnPc molecule remains relatively constant with varying EF values. In contrast, the E_g_ values for XH_3_/MnPc complexes exhibit changes: they increase as the positive EF rises, reaching 1.22, 1.38, and 1.38 eV for NH_3_/MnPc, PH_3_/MnPc, and AsH_3_/MnPc complexes, respectively, at EF = 0.514 V/Å. Conversely, the E_g_ values decrease with an increasing negative EF, reaching 1.18, 1.22, and 1.23 eV for NH_3_/MnPc, PH_3_/MnPc, and AsH_3_/MnPc complexes, respectively, at EF = −0.514 V/Å. This suggests that the electrical conductivity (σ) of the XH_3_/MnPc complexes is influenced by the magnitude and direction of the applied EF. Additionally, the electrical sensitivity (ΔE_g_) dependency on the EF was investigated using the following equation:

(2) $$\Delta E_{g}=\frac{E_{g}({XH}_{3}/MnPc)-E_{g}(MnPc)}{E_{g}(MnPc)}\times 100$$

In Figure 16b, at EF = 0.0 V/Å, the ΔEg values were observed to be −13.68%, −8.43%, and −7.21% for NH_3_/MnPc, PH_3_/MnPc, and AsH_3_/MnPc complexes, respectively. This indicates that the adsorption of XH_3_ molecules leads to a decrease in the E_g_ value for the MnPc molecule, accompanied by an increase in σ. Furthermore, the MnPc molecule exhibited greater sensitivity to NH_3_ compared to PH_3_ and AsH_3_ molecules. Additionally, the influence of the electric field is evident in the ΔE_g_ values: increasing the positive EF reduces the magnitude of ΔE_g_, making it less negative. Conversely, increasing the negative EF intensifies the magnitude of ΔE_g_, making it more negative. Therefore, a positive electric field diminishes the sensitivity, whereas a negative electric field enhances the sensitivity of the MnPc molecule to XH_3_ molecules.

It is noteworthy that the effect of the electric field on the UV-vis spectrum of the XH_3_/MnPc complexes was investigated, but no considerable effect was observed.

## 3. Methods

The interaction of XH_3_ gases onto the MnPc molecule is investigated utilizing DFT [43] calculations at the B3LYP/6-311+g(d,p) level of theory. D3, Grimme’s dispersion that considers the long-range interactions, is considered [44,45]. Yang et al. [46] state that B3LYP gave good performances for metal phthalocyanines. Additionally, B3LYP is utilized for transition metal-doped porphyrins [31,47], and the 3D transition metal complexes for hexaazabipy H2 and gave reliable results [48]. Geometrical optimizations to obtain the most energetically stable structures for free XH_3_ gases, MnPc bare molecule, and XH_3_/MnPc complexes are accomplished. The UV-vis absorption spectra for Pc and MnPc molecules, as well as the XH_3_/MnPc complexes, are estimated by Time-Dependent DFT (TD-DFT) calculations. To cover all the expected electronic transitions in the investigated range (0–1000 nm), a satisfactory number of excited states (n = 15) was projected. To estimate the relative stability of the Pc and MnPc molecules, the average binding energy for each atom (E_b_) was estimated according to Equation (3) [39]. The more negative the E_b_ value, the more stable the molecule.

(3) $$E_{b}=\frac{1}{n}\left(E_{molecule}-\sum_{i=1}^{n}E_{i}\right)$$

where n is the total number of atoms of the molecule, $E_{molecule}$ is the total energy of the molecule-optimized structure, and $E_{i}$ is the single atom’s total energy. The relative chemical reactivity of molecules is estimated according to the following parameters: the HOMO-LUMO energy gap (E_g_), the ionization potential (IP), the chemical potential (µ), hardness (η), and electrophilicity (ω). The high reactive molecule is characterized by low values of E_g_, IP, µ, and η and high value of ω [49,50,51]. 

The IP is evaluated via Koopman’s approximation [49,50]

(4) $$IP\;\approx -E_{HOMO}$$

µ, η, and ω are evaluated by Equations (5)–(7), respectively [50,51].

(5) $$µ\approx -\frac{1}{2}\;\left(E_{HOMO}+E_{LUMO}\right)$$

(6) $$\eta \approx \frac{1}{2}\;\left(E_{LUMO}-E_{HOMO}\right)$$

(7) $$\omega \approx \frac{{µ}^{2}}{2\eta }$$

The strength of the XH_3_-MnPc interaction is judged in terms of the adsorption energy (E_ads_), which is assessed by Equation (8). The strong XH_3_-MnPc interaction is accompanied by more negative E_ads_ values.

(8) $$E_{ads}=E_{XH_{3}/MnPc}-{(E}_{MnPc}+E_{XH_{3}})$$

$E_{XH_{3}/MnPc}$, $E_{MnPc}$, and $E_{XH_{3}}$ are the total energies of the XH_3_/MnPc complex, MnPc bare molecule, and free XH_3_ gases, respectively. The charge density difference (Δρ) for the XH_3_/MnPc complex is calculated as follows:

(9) $$\Delta \rho =\rho_{XH_{3}/MnPc}-{(\rho }_{MnPc}+\rho_{XH_{3}})$$

$\rho_{XH_{3}/MnPc}$, $\rho_{MnPc}$, and $\rho_{XH_{3}}$ are the charge densities for the XH_3_/MnPc complex, MnPc bare molecule, and free XH_3_ gases, respectively.

Thermodynamic calculations are performed in the range of T = 300–800 K to evaluate the enthalpy difference (ΔH) and Gibbs free energy difference (ΔG) for the XH_3_/MnPc complexes by Equations (10) and (11), respectively [52].

(10) $$\Delta H=H_{XH_{3}/MnPc}-{(H}_{MnPc}+H_{XH_{3}})$$

(11) $$\Delta G=G_{XH_{3}/MnPc}-{(G}_{MnPc}+G_{XH_{3}})$$

where $H_{XH_{3}/MnPc}$, $H_{MnPc}$, and $H_{XH_{3}}$ are the enthalpies while $G_{XH_{3}/MnPc}$, $G_{MnPc}$, and $G_{XH_{3}}$ are the Gibbs free energies for the XH_3_/MnPc complex, MnPc bare molecule, and free XH_3_ gases, respectively. The thermodynamic adsorption equilibrium constant (K_ads_) is a crucial adsorption factor. A K_ads_ value higher than 100 is essential for the sorbent to be a useful application. K is evaluated by Equation (12) [18].

(12) $$K_{ads}=e^{-\frac{\Delta G}{RT}}$$

where R is 8.314 J.mol^−1^.K^−1^.

The adsorption behavior under the influence of an external static electric field (EF) was investigated. The EF was applied from −0.514 to 0.514 V/Å with a step of 0.125 V/Å (0.0025 au) along the axis, which was perpendicular to the plane of the MnPc molecule. Gaussian 09 software [53] was used for all calculations. GaussSum 3.0 was used to show the density of states (DOS) for the structures under investigation [54]. To estimate the atomic charges for the structures under examination, a thorough natural bond orbital (NBO) analysis was carried out using the NBO program version 3.1 [55]. A Quantum Theory of Atoms in Molecules (QTAIMs) examination was performed utilizing the Multiwfn 3.7 software package [56].

## 4. Conclusions

An investigation into the interaction of XH_3_ gases (X = N, P, As) with the MnPc molecule, employing DFT calculations and external electric field modulation, has provided valuable insights into the adsorption behavior and its consequences on the electronic and optical properties of MnPc. The confirmed chemical adsorption of XH_3_ onto MnPc, supported by QTAIM analysis indicating partially covalent bonds, underscores the relevance of this study in elucidating the nature of the XH3/MnPc interaction.

The significant reduction in the energy gap (E_g_) of MnPc upon XH_3_ adsorption, accompanied by the observed blue shift in the UV-vis spectrum, not only reveals the sensitivity of MnPc to XH_3_ molecules but also suggests its potential application as a visual sensor. The thermodynamic calculations establish the exothermic nature of the interactions, with NH_3_/MnPc emerging as the most stable complex. The temperature-dependent stability of NH_3_/MnPc complexes adds a nuanced understanding of the dynamics of the adsorption process.

Moreover, the influence of an external electric field on the adsorption energy (E_ads_) highlights the tunability of MnPc’s sensitivity to XH_3_ gases. The magnitude of the applied electric field plays a pivotal role, emphasizing the importance of external factors in tailoring the adsorption characteristics.

In conclusion, this study not only contributes to the fundamental understanding of XH_3_/MnPc interactions at a molecular level but also opens up avenues for potential applications in sensor technologies. The versatility of MnPc, demonstrated through its responsiveness to external stimuli and the dynamic nature of the adsorption process, suggests promising prospects for its utilization in sensing applications. Further exploration in this direction could lead to the development of innovative materials for gas sensing and related technologies.

## Figures and Tables

**Table 1:** Electronic properties of Pc and MnPc. HOMO and LUMO energy levels (eV) for α and β spins, HOMO-LUMO gap (E_g_, eV), average binding energy per atom (E_b_, eV), NBO charges (Q, e), ionization potential (IP, eV), chemical potential (µ, eV), hardness (η, eV), electrophilicity (ω, eV), and dipole moment (D, Debye).

	Pc	MnPc
HOMO (α)	−5.305	−5.242
LUMO (α)	−3.185	−3.117
HOMO (β)	−5.305	−4.798
LUMO (β)	−3.185	−3.410
E_g_	2.120	1.388
E_b_	−5.402	−5.474
Q_TM_	-	0.954
IP	5.305	4.798
µ	4.245	4.104
η	1.06	0.694
ω	8.499	12.134
D	0.021	0.010

**Table 2:** Adsorption properties of XH_3_ (X = N, P, As) on MnPc. Adsorption energies (E_ads_, eV), HOMO and LUMO energy levels (eV) for α and β spins, HOMO-LUMO gap (E_g_, eV), NBO charges (Q, e), and dipole moment (D, Debye).

	Mode 1			Mode 2		
	NH_3_/MnPc	PH_3_/MnPc	AsH_3_/MnPc	NH_3_/MnPc	PH_3_/MnPc	AsH_3_/MnPc
E_ads_	−0.777	−0.414	−0.370	−0.307	−0.340	−0.344
d_X-Mn_	2.333	2.786	2.954	2.995	3.542	3.612
HOMO (α)	−5.079	−5.136	−5.163	−5.285	−5.250	−5.229
LUMO (α)	−2.984	−3.035	−3.058	−3.176	−3.133	−3.111
HOMO (β)	−4.427	−4.582	−4.628	−4.794	−4.781	−4.755
LUMO (β)	−3.230	−3.312	−3.340	−3.459	−3.418	−3.396
E_g_	1.196	1.271	1.288	1.335	1.363	1.359
Q_M_	0.827	0.830	0.767	0.988	0.880	0.898
$Q_{XH_{3}}$	0.177	0.324	0.226	0.084	0.100	0.101
D	3.130	2.083	1.576	0.949	0.228	0.213

**Table 3:** The estimated topological parameters. Electron densities (ρ), Laplacian of charge density (∇^2^ρ), kinetic electron density (G(r)), potential energy density (V(r)), and energy density (H(r)). All units are in au.

Adsorption Mode	Complex	BCP	ρ	∇^2^ρ	G(r)	V(r)	H(r)	−G(r)/V(r)
1	NH_3_/MnPc	N–Mn	0.045	0.171	0.045	−0.047	−0.002	0.952
	PH_3_/MnPc	P–Mn	0.032	0.064	0.021	−0.026	−0.005	0.813
	AsH_3_/MnPc	As–Mn	0.025	0.046	0.015	−0.018	−0.003	0.833
2	NH_3_/MnPc	N–Mn	0.013	0.030	0.008	−0.009	−0.001	0.897
	PH_3_/MnPc	P–Mn	0.008	0.020	0.005	−0.004	0.000	1.097
		H–N	0.007	0.019	0.004	−0.004	0.001	1.155
	AsH_3_/MnPc	As–Mn	0.007	0.019	0.004	−0.004	0.000	1.109
		H–N	0.007	0.017	0.004	−0.003	0.001	1.197

## Data Availability

Data are contained within the article.
